# Quantitative Trait Loci and Candidate Genes for Neutrophil Recruitment in Sterile Inflammation Mapped in AXB-BXA Recombinant Inbred Mice

**DOI:** 10.1371/journal.pone.0124117

**Published:** 2015-05-05

**Authors:** Quyen Cheng, Ze’ev Seltzer, Corneliu Sima, Flavia S. Lakschevitz, Michael Glogauer

**Affiliations:** 1 Department of Periodontology, Faculty of Dentistry, University of Toronto, Toronto, Ontario, Canada; 2 Matrix Dynamics Group, Faculty of Dentistry, University of Toronto, Toronto, Ontario, Canada; 3 Centre for the Study of Pain, Faculties of Dentistry and Medicine, University of Toronto, Toronto, Ontario, Canada; Boston University, UNITED STATES

## Abstract

Neutrophil recruitment (NR) to sites of sterile inflammation plays a key role in tissue damage and healing potential of lesions characteristic to non-infectious inflammatory diseases. Previous studies suggested significant genetic control of neutrophil survival, function, and migration in inflammatory responses to endogenous and exogenous stimuli. We have mapped the murine genome for quantitative trait loci (QTLs) harbouring genetic determinants that regulate NR in SI using a murine model of chemically-induced peritonitis. NR was quantified in 16 AXB-BXA recombinant inbred strains and their progenitors, A/J (A) and C57BL/6J (B). A continuous distribution of NR was found among the strains, with parent B showing higher NR and parent A showing lower NR (3.0-fold difference, p=0.05). Within the progeny strains, a 5.5-fold difference in NR was observed between the lowest, BXA1, and the highest responders AXB19 (p<0.001). This data was analyzed using GeneNetwork, which linked NR to one significant QTL on chromosome 12 (Peritoneal Neutrophil Recruitment 1, *PNR1*) and two suggestive QTLs (*PNR2*, *PNR3*) on chromosomes 12 and 16 respectively. Sixty-four candidate genes within *PNR1* were cross-referenced with currently published data, mRNA expression from two NR microarrays, and single nucleotide polymorphism analysis. The present study brings new light into the genetics of NR in response to cell injury and highlights potential candidate genes *Hif1α*, *Fntb*, and *Prkch* and their products for further studies on neutrophil infiltration and inflammation resolution in sterile inflammation.

## Introduction

The innate immune system is a key player in inflammatory responses to microbial invasion and host cell death. Specifically, sterile inflammation (SI) is a critical process in the pathogenesis of chronic conditions triggered and sustained by cell death in the absence of exogenous stimuli combined with a failure to resolve inflammation and restore homeostasis [1]. The initiation of chronic SI is similar to acute ischemic injury of myocardium, traumatic injury and chemotherapeutic-induced tumor death. Like exogenous stimuli, endogenous host-derived factors stimulate early neutrophil infiltration at site of injury. However, recruitment of neutrophils and monocytes is different in the absence of microorganisms suggesting differentially regulated resolution of inflammation. Specifically, toll-like receptors (TLRs) are unlikely the major sensors of cell death in sterile inflammatory responses. Neutrophil recruitment (NR) to sites of sterile cell injury is more dependent on receptor for advanced glycation end products (RAGE) and IL-1R than TLRs compared to recruitment of monocytes [1]. Tissue hypoxia is a major regulator of NR at sites of SI as extensively described in ischemia-reperfusion injury studies. Hypoxia causes activation of transcriptional programs responsible for the turnover ATP released from dying cells to ADP and AMP [2]. Extracellular viable mitochondria, released from necrotic cells, generate ATP that triggers the activation of the inflammasome whose role is critical in the initiation of sterile inflammatory response to tissue injury [1].

The study of inflammatory responses to cell death has gained much attention recently in light of a paradigm shift with regard to therapeutics of uncontrolled inflammation from exclusively anti-inflammatory to anti-inflammatory and pro-resolution agents [3]. Mounting evidence suggests that neutrophil phenotype is critical to initiation of resolution programs [4]. Several chronic diseases characterized by unregulated inflammation present with persistent neutrophil-mediated tissue damage and persistent pro-inflammatory macrophages. Neutrophil hyper-migration plays a key factor in SI characteristic to diseases such as myocardial infarction [5] and rheumatoid arthritis [6], suggesting that tight regulation of NR is required for activation of coordinated resolution programs that re-establish tissue homeostasis. It is therefore currently believed that control of neutrophil infiltration using selective therapeutics that do not impair the host’s ability to fight infections is crucial for limiting tissue damage and promoting resolution of inflammation. An in-depth understanding of NR regulation can identify potential novel therapeutic interventions for neutrophil-mediated disorders. Animal studies have suggested genetic control of neutrophil function during inflammatory responses. For example, experimental inflammation in animal models demonstrated genetic-related differences in NR to the peritoneum [7], blood, lungs, liver, synovial-like cavities, and subcutaneous sites of inflammation [8]. In fact, the complexity of the genetic control of NR is demonstrated through the discovery of multiple regions in genome of rodents harboring genes involved in this process [8,9]. Here, an *in silico* approach was used to map the murine genome for quantitative trait loci (QTLs) harbouring genetic determinants that regulate NR in mice.

Over the past few decades, murine genetic reference populations (GRP) have been useful tools in mapping QTLs affecting polygenetic diseases. One such panel of GRP was derived from reciprocal crosses of inbred mice of the A/J (A) and C57BL/6J (B) parental strains, which resulted in 27 viable, genetically unique and commercially-available AXB-BXA recombinant inbred (RI) strains [10]. The A and B parental strains differ in their susceptibility to over 30 different infectious or chronic diseases and the genetic factors controlling their susceptibility for these diseases is distributed throughout the genome [10]. All 27 AXB-BXA RI strains and their parental strains have been genotyped previously using thousands of markers that identify the parental origin [10]. These properties and resources have provided researchers a powerful tool with which to map QTLs for various traits that contrast in the two parental strains. WebQTL is an internet-based package of statistical genetic software providing an unbiased approach to examine the significance of the linkage between quantifiable traits, such as NR [11]. This program also identifies the genome-wide presence and location of genetic determinants having a significant or suggestive effect on the variability of the trait of interest across the panel of strains used.

The present study aimed at identifying new genes with potential roles in regulation of early NR in SI, with special focus on the importance of early NR for initiation of resolution programs. We found that NR in SI is linked to one significant QTL that we named *PNR1* (Peritoneal Neutrophil Recruitment 1), and two suggestive QTLs, named *PNR2* and *PNR3*. Browsing the murine genome at these QTLs, we identified a few candidate NR genes pertinent to SI that will require validation in future research.

## Methods

### Animals

All procedures conducted were approved by the University of Toronto Animal Care Committee and adhered to the Guide for the Humane Use and Care of Laboratory Animals. Mice from 21 AXB-BXA RI strains (AXB1, AXB2, AXB4, AXB5, AXB8, AXB10, AXB12, AXB13, AXB15, AXB19, AXB24, BXA1, BXA2, BXA4, BXA7, BXA11, BXA12, BXA13, BXA15, BXA24, BXA25) along with the parents, A and B, were obtained from the Jackson Laboratory (Bar Harbor, ME) to serve as breeders. The mice were housed at the Animal Care Facility (University of Toronto) in polycarbonate boxes covered with sterilized pine bedding. The environment was maintained at 21–23°C and 45–55% relative humidity with a 12:12 hour dark:light cycle. The mice were weaned at postnatal day 21 and the males were housed in groups of up to 4 mice per cage. Animals receive chlorinated water and rodent chow *ad libitum* (Harlan Teklad Global Diet # 2018). AXB2, AXB10, BXA12, BXA14 and BXA25 did not produce offspring, thus, the following data reports on NR levels in 16 RI strains. Up to 6 mice/ strain were used from each of the remaining RI strains and parental strains (N = 80; 9–18 weeks old) were used to phenotype the level of NR in response to experimental SI.

### Sodium periodate peritonitis

SI was induced by injection of the chemical irritant sodium periodate (NaIO_4_; 1ml of 5mM, i.p.; Sigma, Oakville, ON, Canada) in phosphate buffered saline (PBS) [12]. The mice were sacrificed 3h after peritoneal injection by CO_2_ asphyxiation and peritoneal exudate was collected by lavage with 10 mL chilled PBS. Following 3 hours of NaIO_4_-induced peritonitis, cells isolated from the peritoneum and mounted onto slides using Cytospin™ 4 Cytocentrifuge (Thermo Fisher Scientific). Greater than 90% of all cells were confirmed to be neutrophils by hematological stain (Diff-Quik, Siemens, Deerfield, IL). Neutrophils were counted by a hemocytometer (Bright-line, Hausser Scientific, Horsham, PA). Four counts were conducted per mouse, averaged, and expressed as a mean ± SEM per strain.

### Heritability estimate

Heritability of NR was estimated using the ratio of inter-strain variance (that is conferred by the allelic variance) over the total variance that combines the allelic and environmental variances [13].

### QTL analysis

QTL mapping is a statistical tool that is used to identify confidence lengths on the DNA that harbour genetic loci controlling the variance in phenotypic levels across inbred strains of rodents. The mean strain NR scores were fed to the WebQTL software (www.genenetwork.org). Using this software, genome-wide interval mapping was carried out using the likelihood ratio statistic (LRS). The LRS describes the relative probability of two different options to explain the observed differences in NR levels among the RI strains. The first option is that the differences in the SDP of a trait under study are linked to the particular DNA sequence. The second option, the null hypothesis, is that there is no linkage between the phenotype and the genotype at a genetic marker locus. An LRS score corresponding to a genome-wide p-value of 0.05 is considered “significant” whereas a p value of 0.63 is considered “suggestive”. For traits having a moderate genetic control over the trait variance the graph shows a few confidence lengths throughout the genome that harbor locus markers with suggestive or even significant LRS values. The LRS score is then converted to the more familiar likelihood of odds (LOD) score by division of 4.61. A confidence interval on the chromosome delimited by the location of locus markers still significant after a 1.0–2.0 drop-off in the LOD score maps the confidence limits of a QTL [14]. Bootstrapping statistical analysis was also employed to support the LRS/ LOD statistics. The higher the bootstrap results, the more confident one can be of the peak location of a QTL observed. Next, a pair-scan model was used to statistically test if two putative QTLs interact to explain the SDP.

### Candidate gene selection

While WebQTL identifies genomic intervals that are associated suggestively or significantly with a phenotype under study, such QTLs typically span lengths that are megabase (Mb) pairs long, which contain up to hundreds of genes. To sift through these lists and identify a few candidate genes that may actually control the variance of the NR SDP, we used the following filtration steps: (1) A literature analysis was carried out using PubMed, National Center for Biotechnology Information (NCBI) as the browsing engine. Each candidate gene was searched using the “AND” function with eleven terms selected to be relevant to NR including “neutrophil”, “neutrophil recruitment”, “neutrophil activation”, “neutrophil infiltration”, “neutrophil chemotaxis”, “innate immunity”, “cell migration”, “actin”, “cytoskeleton”, and “GTPases” [3,15,16]. Article abstracts and/or full text articles were studied and those linking certain gene candidates mapped to identify QTLs to certain cellular functions were flagged as candidates. (2) Genes identified by WebQTL were cross-referenced with two Illumina Mouse-6 v2 Expression BeadChip microarray results from our lab (The microarray data complies with MIAME guidelines, and the data set was deposited at Gene Expression Omnibus (NCBI), accession number GSE43513) [17]. One microarray data set compared the fold change in mRNA gene expression of bone marrow neutrophils to that of neutrophils recruited to the peritoneum following sodium periodate stimulation. The other microarray data set compared gene expression of blood neutrophils to that of peritoneal neutrophils recruited in mice using the same chemical irritant. While peritoneal neutrophil isolation was similar to that described in this paper, details of BM and blood neutrophil isolation can be found in Lakschevitz [17]. Genes located in the QTLs identified in the present study and also in the two gene expression studies were flagged as candidate genes if their absolute fold change of 1.49 or higher and was at a genome-wide significance level of p<0.05. (3) The WebQTL registry has a collection of 4.6x10^6^ SNPs varying between A *vs*. B. This registry was compiled from a number of independent resources including Celera Genomics, Perlegen/NIEHS resequencing project, Wellcome-CTC SNPs, dbSNP, and the Mouse Phenome Database. We used this registry to seek genes in those QTLs having SNPs differing in the genomes of A and B mice, and used this data as one of the criteria to flag candidate genes for NR.

### Statistical analysis

All statistical analyses were carried out using IBM SPSS Statistics (version 20.0). Two-tailed independent t-tests were employed to analyze the mean NR between the two parental strains and of the strains showing the highest and lowest NR counts, AXB19 and BXA1, respectively. Strain data are expressed as strain means ± SEM and strain differences were considered significant at p-values of 0.05 or less.

## Results

### Peritoneal NR in AXB-BXA RI strains and their A, B parental strains

**Fig 1:**
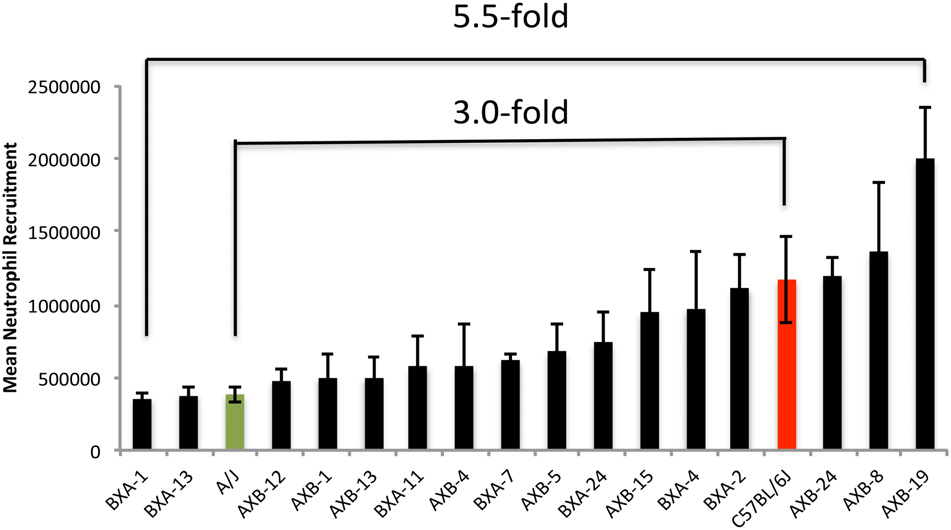
Neutrophil recruitment variation in AXB-BXA RI and the parental mouse strains, A and B (n = 80, up to 6mice/strain). Hemocytometer cell count is expressed as mean number of neutrophil recruited ± SEM. This strain distribution panel shows a 3.0- fold recruitment variation between the 2 parental strains (p = 0.05) and 5.5-fold between the lowest and highest responders (p<0.001).

The neutrophil’s involvement and contribution to SI depends on its ability to respond to *in vivo* cell death and migrate to the site of injury. Peritoneal NR using synthetic irritants has been used as an effective model of inducing a localized SI response. Comparing the mean NR scores (Fig 1) showed a 3.0-fold difference (p = 0.05) between the values of the two parental strains (A = 3.84X10^5^ ± 5.24 X10^4^ cells *vs*. B = 1.17 X10^6^ ± 2.95 X10^5^ cells). However, a considerably stronger contrast in the peritoneal NR was found in the SDP of the RI strains, with a 5.5-fold difference (p<0.001) between the lowest responder BXA1 (3.61 X10^5^ ± 3.64 X10^4^ cells) and highest responder AXB19 (1.99 X10^6^ ± 3.53 X10^5^ cells). These strain values surpassed those of the parental strains compatible with a heterosis model.

### Heritability

Heritability for NR was estimated to be 0.58, suggesting that over half the variation in this phenotype can be attributed to genetic control. This indicates that NR in mice is ‘adequately heritable’ to be used in QTL analysis.

### Significant QTL on Chr 12 (*PNR1*)

**Fig 2:**
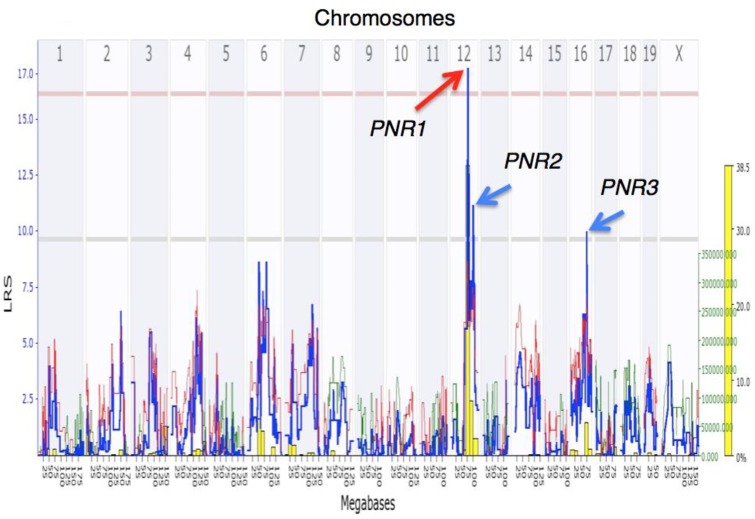
Genome-wide interval mapping of NR in AXB-BXA RI mice and their A and B parents (n = 80, up to 6mice/strain). The significant Likelihood Ratio Statistic (LRS) values associated with marker loci on Chr 12 peaking at an LRS = 17.5 (LOD = 3.8) delimit a QTL (*PNR1*) at a confidence length spanning from 72.81 to 78.28 Mb. The analysis mapped two additional suggestive QTLs: *PNR2* maps to Chr 12 and is associated with an LRS = 11.0 (LOD = 2.4, p < 0.63), spanning from 87.0–101.6 Mb, and *PNR3* maps to Chr 16, associated with an LRS = 9.7 (LOD = 2.1, p < 0.63) and spanning from 56.5–75.0 Mb. The overlaying histogram (yellow columns) shows results of the bootstrapping statistical analysis (in % of the 2000 reiterations, see text). The contribution to the variance conferred by alleles from parent B (red line) or A (green line) at each marker locus along the genome is associated with values measured by the right Y axis (green font).

The interval mapping software, followed by a 2.0-LOD drop-off, identified a significant QTL on Chr 12 spanning a confidence interval of 5.47 Mb (from 72.81 Mb to 78.28 Mb; LRS = 17.52, LOD = 3.8)) that we nicknamed *PNR1* (Peritoneal Neutrophil Recruitment 1) (Fig 2). This QTL harbors 64 genes (Table 1).

**Table 1:** Genes from *PNR1*.

Gene Symbol			
Daam1	Six6	Dbpht2	Akap5
Gdap5	Six1	4930442G10Rik	A430103D13Rik
Gpr135	Six4	Kcnh5	Zbtb25
2810055F11Rik	Mnat1	Rhoj	6330522J23Rik
2310047L11Rik	D630012G11Rik	C230007H23Rik	Zbtb1
1200003C05Rik	Trmt5	Gphb5	4930426I24Rik
4930403N07Rik	Slc38a6	Ppp2r5e	Hspa2
Rtn1	D830013O20Rik	Wdr89	Plekhg3
Lrrc9	Tmem30b	LOC665931	Spnb1
1810048J11Rik	2210039B01Rik	Sgpp1	Churc1
2900009J20Rik	Prkch	Syne2	Gpx2
Dhrs7	Hif1a	A930027M08Rik	Rab15
5033425G24Rik	Snapc1	Esr2	Fntb
Ppm1a	9630050P21Rik	Tex21	Max
5730409N16Rik	Syt16	Mthfd1	9530018F02Rik
Six6os1	1700086L19Rik	Gm70	1700123J03Rik

Full list of 64 candidate genes located in the *PNR1* QTL on Chr 12. *PNR1* spans a confidence interval of 5.47 Mb (from 72.81 Mb to 78.28 Mb).

### Suggestive QTLs identified on Chr 12 (*PNR2*) and Chr 16 (*PNR3*)

Using the same NR data set and a 1.0-LOD drop-off, two suggestive QTLs were additionally mapped, one on Chr 12 spanning a length of 18 Mb (from 87.0–101.6 Mb; LRS = 11.1, LOD = 2.4), containing 130 genes (nicknamed *PNR2*; S1 Table *)*. The second suggestive QTL, nicknamed *PNR3*, and associated with an LRS = 9.7 (LOD = 2.1), was mapped to Chr 16, spanning 18.5 Mb (from 56.5–75.0 Mb), containing 96 genes (S3 Table). Microarray analyses were conducted where *Batf*, *Zc3h14*, and *6430527G18Rik* from *PNR2* (S2 Table) and *St3gal6* and *2610528E23Rik* from *PNR3* (S4 Table) showed significant fold changes with both expression data sets. It has been previously shown that *St3gal6* is directly linked to NR in mice [18]. *Batf* was suggested to promote allergic inflammation via the activation of T helper 9 cells [19] however has no existing linkage to neutrophils. The remaining genes showed no significance with our key terms.

### Gene candidates for *PNR1*

The 64 genes harbored within *PNR1* were sifted using the filters (1) having literature significance using key terms described above, (2) showing significant fold change in NR gene expression studies, and (3) having SNPs differing between the parental strains. Genes fitting criteria (1) and/ or (2) are listed in Table 2. Six genes have demonstrated links to neutrophil function (*Daam1*, *Ppm1a*, *Hif1α*, *Syne2*, *Fntb*, *Max*). Seven additional genes (*Prkch*, *Dhrs7*, *Rhoj*, *Sgpp1*, *Esr2*, *Akap5*, *Gpx2*) have been described in other cell systems to be involved in cellular movement. Genes outlined by WebQTL were cross-referenced with the gene lists in the two microarray experiments (Table 2). Five genes from the 64 *PNR1* gene list (*Hif1α*, *Prkch*, *Fntb*, *1200003C05Rik*, *Snapc1*) were significant on both microarrays, highlighting that their mRNA expression was altered during transit between compartments within the mouse. Three of the genes (*Hif1α*, *Fntb*, and *Prkch)* that were significant in the literature search were also differentially expressed in both microarrays. SNP analysis was conducted between the parents for short-listed genes (Table 3). The top three genes revealed numerous SNPs, however, the vast majority were either translationally synonymous or within the introns. *Fntb* and *Prkch* presented with differences in the 3’ UTR (untranslated region). This region has been discovered to potentially have a powerful effect on mRNA stability, localization, and ultimately gene expression [20]. Minor SNP differences were revealed between parents for *Hif1α*. All three were selected for further analysis.

**Table 2:** Short-listed candidate genes located in the *PNR1* QTL on Chr 12.

Index	Symbol	Gene	Microarray	Microarray	Literature Significance^#^
			BM vs. PE	Blood vs. PE	
			FC*	FC*	
1	***Hif1a***	**Hypoxia inducible factor 1, alpha subunit**	4.72	7.43	[29]
2	***Prkch***	**Protein kinase C, eta**	2.20	2.37	[23]
3	***Fntb***	**Farnesyltransferase, CAAX box, beta**	-1.91	-3.63	[22]
4	*Dhrs7*	Dehydrogenase/reductase SDR family member 7	-2.79		[34]
5	***1200003C05Rik***	**RIKEN cDNA 1200003C05 gene**	-1.65	-1.71	
6	*Ppm1a*	Protein phosphatase 1A	-1.886		[35]
7	***Snapc1***	**snRNA-activating protein complex subunit 1**	1.493	1.58	
8	*Max*	Max protein		1.79	[36]
9	*Mnat1*	Menage a trois 1/ cyclin H assembly factor		-1.55	
10	*Daam1*	Disheveled-associated activator of morphogenesis			[37]
11	*Rhoj*	Ras homolog family member J			[38]
12	*Sgpp1*	Sphingosine-1-phosphate phosphatase 1			[39]
13	*Syne2*	Spectrin repeat containing, nuclear envelope 2; Nesp2g			[40]
14	*Esr2*	Estrogen receptor 2 (beta)			[41]
15	*Akap5*	A kinase (PRKA) anchor protein 5			[42]
16	*Gpx2*	Glutathione peroxidase 2			[43]

Sixteen genes were prioritized through a selective literature search, inclusion in 2 NR gene expression studies, and presence of SNPs differing in the parental strains.

(-) indicates mRNAs were downregulated as PMNs transit from BM to PE and Blood to PE. BM—Bone Marrow; PE—Peritoneum; FC—Fold Change.

* FC significant at p<0.05.

^#^Using PubMed, each candidate gene was searched using the “AND” function with 11 terms selected to be relevant to NR including “neutrophil”, “neutrophil recruitment”, “neutrophil activation”, “neutrophil infiltration”, “neutrophil chemotaxis”, “innate immunity”, “cell migration”, “actin”, “cytoskeleton”, and “GTPases”. Bolded genes show significance with both microarray data sets.

**Table 3:** SNPs varying between the parental strains A and B for filtered genes in the significant *PNR1* QTL.

Index	Symbol	SNP Count	ID	Mb	Domain	Function	Source	Strains	
								A B	
1	Hif1a	197	wt37-12-75033404	75.033404	Exon 6	Synonymous/ protein coding	Sanger/UCLA	C	T
2	Prkch	292	wt37-12-74878483	74.878483	Exon	3’UTR	Sanger/UCLA	C	T
			wt37-12-74878598	74.878598	Exon	3’UTR	Sanger/UCLA	T	G
3	Fntb	150	wt37-12-77988719	77.988719	Exon 7	Synonymous/ protein coding	Sanger/UCLA	T	C
			wt37-12-78021734	78.021734	Exon	3’UTR	Sanger/UCLA	G	C
4	Dhrs7	4	wt37-12-73751463	73.751463	Exon	3’UTR	Sanger/UCLA	T	C
			rs29173979	73.754202	Exon 5	Synonymous/ protein coding	dbSNP	A	G
5	1200003C05Rik	6			Intron	Non-splice site	Sanger/UCLA, dbSNP		
6	Ppm1a	14			Intron	Non-splice site	Sanger/UCLA, dbSNP		
7	Snapc1	34	wt37-12-75070620	75.070620	Intron	Splice site	Sanger/UCLA	C	T
			wt37-12-75072923	75.072923	Exon 6	Non-synonymous/ protein coding	Sanger/UCLA	C	T
			wt37-12-75073538	75.073538	Exon 7	Synonymous/ protein coding	Sanger/UCLA	A	T
			wt37-12-75085002	75.085002	Exon	3’UTR	Sanger/UCLA	A	G
			wt37-12-75085537	75.085537	Exon	3’UTR	Sanger/UCLA	A	G
8	Max	6			Intron	Non-splice site	Sanger/UCLA		
9	Mnat1	325	wt37-12-74373930	74.373930	Exon	3’UTR	Sanger/UCLA	T	C
			wt37-12-74374446	74.374446	Exon	3’UTR	Sanger/UCLA	G	A
			wt37-12-74374838	74.374838	Exon	3’UTR	Sanger/UCLA	G	C
10	Daam1	326	wt37-12-73091262	73.091262	Exon	3’UTR	Sanger/UCLA	A	T
			wt37-12-73092065	73.092065	Exon	3’UTR	Sanger/UCLA	T	C
			wt37-12-73092404	73.092404	Exon	3’UTR	Sanger/UCLA	G	T
			wt37-12-73092739	73.092739	Exon	3’UTR	Sanger/UCLA	A	G
11	Rhoj	6			Intron	Non-splice site	Sanger/UCLA		
12	Sgpp1	4			Intron	Non-splice site	Sanger/UCLA		
13	Syne2	24			Intron	Non-splice site	Sanger/UCLA		
14	Esr2	8			Intron	Non-splice site	Sanger/UCLA		
15	Akap5	0			Intron	Non-splice site	Sanger/UCLA		
16	Gpx2	3			Intron	Non-splice site	Sanger/UCLA		

SNPs of potential significance are listed; the remaining are found in introns at non-splice sites.

HIF1 is a highly conserved transcription factor complex, which plays a key role in oxygen homeostasis. It is a heterodimer composed of HIF1α and HIF1β subunits. In normoxia, HIF1α is targeted for destruction by an ubiquitin ligase complex. In hypoxia, this destruction is inhibited and HIF1α is allowed to accumulate and translocate to the nucleus where it binds the constitutively expressed HIF1β. This complex regulates the transcription of a number of genes involved in response to hypoxia including cell proliferation, energy metabolism, erythropoiesis, angiogenesis, vascular remodeling [21].

The second candidate gene that was highlighted by our selection criteria (*Fntb)* encodes for farnesyltransferase, CAAX box, beta. *Fntb* is involved in post-translational modification of a number of proteins known to be involved in NR including IL-8 priming of neutrophils during activation of the respiratory burst [22].

The third candidate gene (*Prkch*), encoding for protein kinase C eta, is another important candidate NR gene in *PNR1*. This PKC isoform has been shown to be involved in immune cell oxidative stress [23] and its polymorphism has been linked to both rheumatoid arthritis [24] and cerebral infarction [23].

### Epistasis controlling high NR

**Fig 3:**
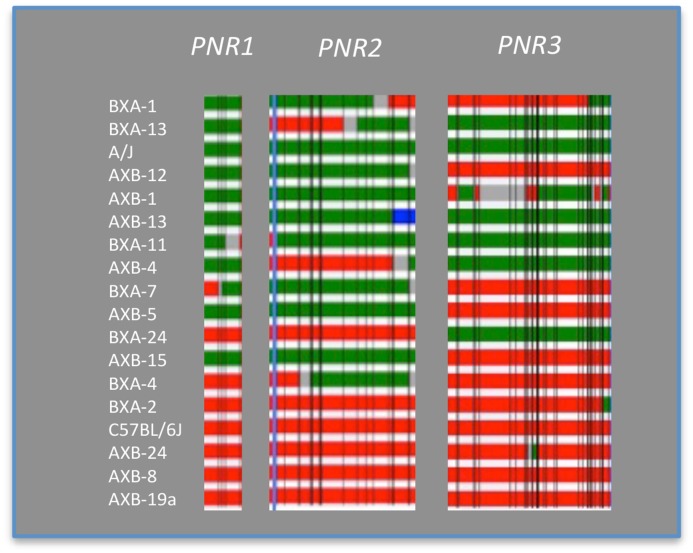
Genotype analysis for epistatic effect between pairs of QTLs. Genotype at each QTL was mapped for 18 mouse strains. Red bar represents homozygous for B6 genotype (B/B), green bar represents homozygous for A/J genotype (A/A), blue bar indicates heterozygous alleles (A/B), gray bars are regions not yet genotyped, and thin perpendicular black lines show the location of marker loci.

**Fig 4:**
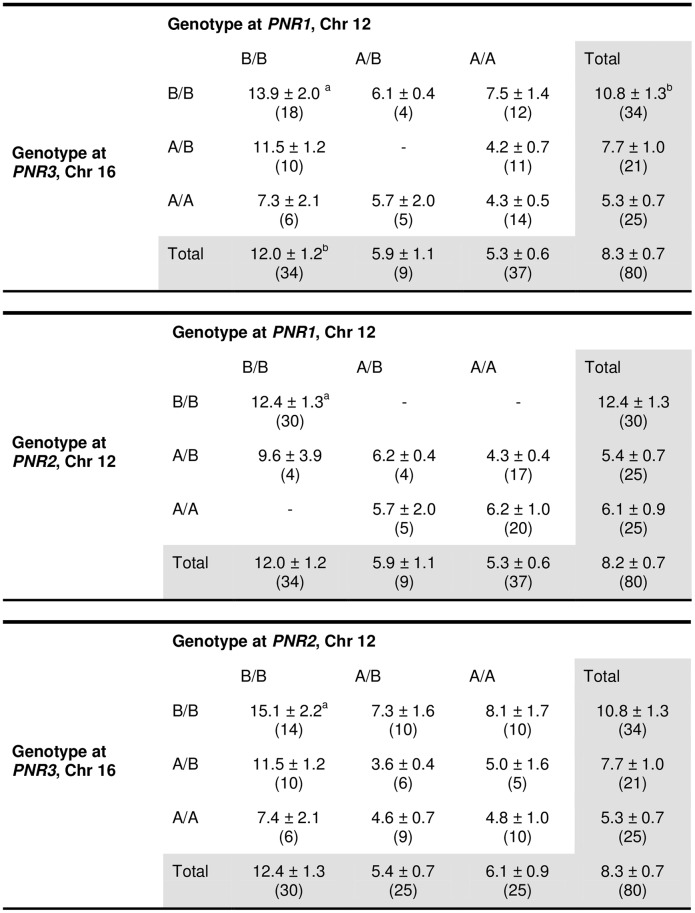
Mean NR ± SEM values are shown for each genotype class (x 105). Number of mice is shown in brackets. ^a^In the *PNR1* and *PNR2* QTLs significantly higher mean NR values were associated with animals carrying homozygous B alleles than A alleles (p<0.001).

**Fig 5:**
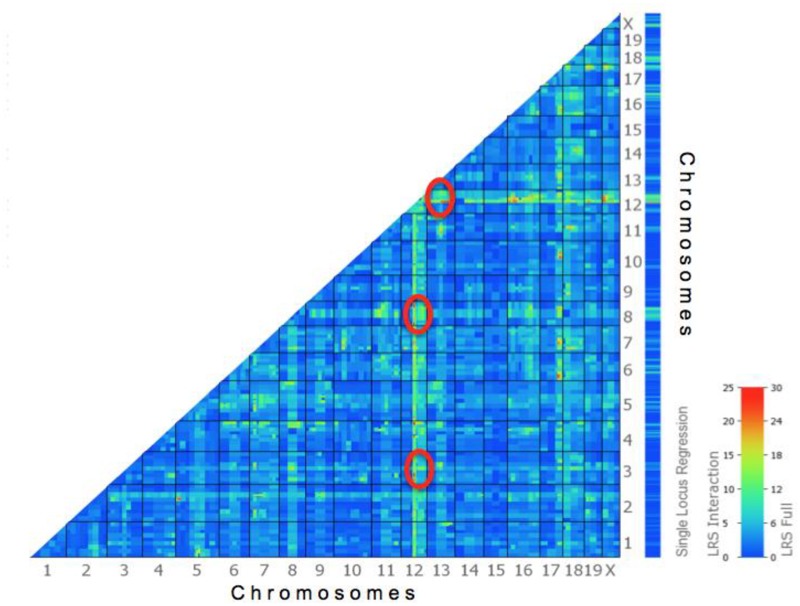
Heat map output of the pair-scan analysis showing the strength of the interaction between any two marker loci on the genome. ‘Warmer’ colors indicate a high correlation with red being the strongest, and ‘cooler’ colors indicate a low correlation. Markers on Chrs 3, 8, and 13 associated with the NR trait (denoted by the red ovals) show a higher epistatic interaction with *PNR1* markers on Chr 12.

A QTL locus is B/B if only parent B contributed the SNPs (red), A/A if only parent A contributed (green), and A/B if both parents contributed the SNPs (Fig 3). Interactions between QTL pairs *PNR1 vs*. *PNR3*, *PNR1 vs*. *PNR2* and *PNR2 vs*. *PNR3* (Fig 4) were analyzed by comparing the nine genotypic combinations between each pair (method adopted from [9]). Similar trends were seen in all three comparisons. Indeed, when two QTLs were of the B/B genotype, NR was significantly higher than those with both A/A genotype (Fig 4; p<0.001). However, epistasis could not be concluded here since having B/B genotype at two QTLs was not statistically significant from other genotype combinations other than A/A homozygous. The absence of a significant epistatic interaction between the QTLs on Chr 12 and 16 was also determined using pair-scan analysis (Fig 5, Table 4). However, the top 20 pairs of intervals suggested epistatic interactions between Chr 12 with Chrs 13, 4, and 8, and Chr 2 with 5. These results must be interpreted with caution since pair-scan is a very powerful and sensitive tool but requires a high sample size (www.genenetwork.org). Although the interaction between these highlighted QTLs are not conclusive, having the B allele at PNR1-3 is associated with higher NR (Figs 3 and 4)[25]. This suggests a contribution of these loci, in part, in controlling NR variance.

**Table 4:** Top 20 pairs of interacting loci from pair-scan analysis.

Locus 1				Locus 2		
	Flanking markers				Flanking markers	
Chr	Proximal	Distal	LRS Full	Chr	Proximal	Distal
12	rs13481533	rs13481542	35.180	13	rs3688563	gnf13.083.500
2	rs13476719	rs6276129	33.549	5	rs3671575	rs6349956
2	rs13476708	rs4223406	33.446	5	rs3671575	rs6349956
4	UT_4_147.232882	rs4224947	33.384	12	rs13481533	rs13481542
2	rs13476719	rs6276129	33.356	5	rs3671575	rs6349956
2	rs13476708	rs4223406	33.170	5	rs3671575	rs6349956
12	rs3685417	rs13481531	33.087	13	rs3688563	gnf13.083.500
4	rs3720325	D4Mit127	32.073	12	rs13481533	rs13481542
2	rs13476719	rs6276129	31.854	5	rs3671575	rs6349956
2	rs13476708	rs4223406	31.602	5	rs3671575	rs6349956
3	rs3674296	rs13477126	31.459	12	rs13481533	rs13481542
12	rs13481533	rs13481542	30.791	13	rs3719701	rs13481949
12	rs13481533	rs13481542	30.739	13	rs6288319	rs6208142
12	rs13481533	rs13481542	30.701	13	rs3719701	rs13481949
12	rs13481533	rs13481542	30.516	13	rs3719701	rs13481949
3	rs3674296	rs13477126	30.316	12	rs13481533	rs13481542
12	rs13481533	rs13481542	30.255	13	rs3719701	rs13481949
12	rs13481533	rs13481542	30.049	13	rs13481953	gnf13.095.805
12	rs13481533	rs13481542	30.048	13	rs3088752	rs4230027
12	rs13481533	rs13481542	29.946	13	rs6288319	rs6208142
8	CEL-8_7689226	gnf08.006.700	29.739	12	rs13481533	rs13481542

### Discussion

Investigation of NR in response to tissue injury is critical for understanding inflammation resolution mechanisms and designing pro-resolution therapeutics for non-resolving inflammation. Neutrophil infiltration at sites of SI seems to be a rate limiting step for the course of resolution both quantitatively and qualitatively [26]. The present study investigated the genetic aspects of NR early during SI. We identified 3 QTLs (*PNR1-3)* that are linked to NR in SI. They appear to act independently of each other to affect variation in the NR levels across the AXB-BXA RI strain panel used. Candidate genes prioritized in *PNR1* may affect NR levels by conferring cytoprotection in inflammatory sites and may therefore be involved in various immune cell functions. The strongest candidate is *Hif1α*, encoding for HIF1α, the alpha subunit of Hypoxia Inducible Factor 1, which may control early NR and survival into injured tissues during SI.

NR was quantified using a NaIO_4_-induced peritonitis model. The cellular effects of NaIO_4_ are mediated in part by oxidation of cell surface molecules to generate of free aldehydes in a process that results in cellular necrosis [27]. NR differed between the parental strains by 3.0-fold, where the A strain was the weaker NR-responder to the irritant. If this were a Mendelian trait, one would have expected to see a 1:2:1 distribution pattern in NR levels across tested RI strains. However, trait levels showed a continuous distribution implying a polygenetic inheritance. This finding complicates the phenotypic control of neutrophil migration beyond a simple gene-by-environment interaction.

The distribution of NR values of the inbred mice and their parents enabled us to map three QTLs, named *PNR1*, *PNR2*, and *PNR3*. To our knowledge, this is the first study to identify, in an unbiased method, QTLs regulating NR into the peritoneum of mice during SI. Previous genetic studies investigating neutrophil migration have been conducted, however, these studies used different animal models, inflammatory sites, and infection induction methods when compared to our study [8,9]. Using an LPS-induced model of endotoxic shock, Matesic *et al*. studied neutrophil migration into hepatic sinusoids and mapped 2 QTLs on Chrs 5 and 13 (named *Hpi1* and *Hpi2* respectively), controlling high neutrophil infiltration [9]. Lariagone *et al*. on the other hand mapped 3 QTLs: *Cia4* on Chr 7, *Cia6* on Chr 8, and *Cia7* on Chr 2, that regulate migration of neutrophils into synovial-like inflamed tissues in a rat model of rheumatoid arthritis [8,28]. It is noteworthy that none of these corresponds to the mapped QTLs identified in the present study.

Similar to Lariagone *et al*., our study focused on neutrophil recruitment in SI in contrast to recruitment triggered by infectious stimuli. Furthermore, a pair-scan analysis revealed a relationship between seven of the nineteen autosomal chromosomes in the present study (Table 4). The resulting interacting loci were investigated and genes in proximity to the markers were discovered. However, a comparison of these paired sequences with QTLs discovered in Matesic *et al*. and Lariagione *et al*. did not reveal any synteny between sequences or genes. Despite generally similar triggering pathways in sterile and microbial-induced inflammation, specific receptor pathways to endogenous stimuli are of importance for atherosclerosis and rheumatoid arthritis [1]. This further suggests that NR is a complex trait that may be regulated by unique species-specific, site-specific and irritant-specific gene pools. Therefore understanding the differences and commonalities will help elucidate unique pathways involved in inflammatory processes to develop therapy targets.

The initial step for NR during inflammation is largely dependent on the binding of selectins to their glycan ligands. Sialyltransferases are a family of enzymes involved in the generation of selectin ligands. Interestingly in a recent study by Yang et al., it was shown that mice with sialyltransferase 6 (*St3gal6*, PNR3, S4 Table) knockout showed deficient NR to the peritoneum under experimental sterile inflammation conditions [18]. In fact, this effect does not extend to monocyte recruitment. It was concluded that *St3gal6* affects neutrophil rolling and is critical for NR during inflammation. *St3gal6* appears to be a strong candidate for affecting PNR3 during NR as it is significant on both microarray assays as well as showing literature linkage to NR in vivo.

Inspection of the genotypes at the 3 QTLs *PNR1-3* shows that AXB24, AXB8, AXB19 carry the same genotype as their B parental strain but reached NR values beyond that of B. The heterosis seen here suggests a possible epistatic effect between these 3 QTLs and genetic determinants elsewhere on the genome. As well, offspring with mixed genotype (A/B) showed lower NR than the original B6 parent. One possibility is that the B genes at these particular loci have small or no effect on NR and/or alleles originating from the A parent have an important role in NR. QTL interaction analysis by way of the pair-scan test did not show an epistatic interaction between the three QTLs. This may be a true effect, indicating these loci operate alone. Nevertheless, it can be concluded from these observations that the QTLs identified have the ability to influence NR by way of an enhancing effect when carrying the B genotype.

Strain differences in NR is linked to 64 genes delimited in the 5.47cM interval of the *PNR1* QTL. The prime candidate is *Hif1α*, the alpha subunit of Hypoxia Inducible Factor 1. The protein product, HIF1α, is a known essential factor for myeloid cell activation and function in an inflammatory or necrotic environment in response to hypoxia [29]. Both sterile and pathogen-induced inflammation are characterized by altered tissue homeostasis including excess extracellular ROS, release of pro-inflammatory cytokines, and significant phagocyte infiltration resulting in a highly hypoxic environment [30]. From the microarray analyses, the mRNA expression levels of *Hif1α* was up-regulated in peritoneal neutrophils compared to neutrophils isolated from the bone marrow and blood. In fact, Sparkenbaugh *et al*. demonstrated decreased hepatic neutrophil infiltration and reduced plasma concentration of IL-6 and keratinocyte chemoattractant during acetaminophen hepatotoxicity in *Hif1α* ^***-/-***^ mice, associated with overall less severe tissue injury by 24h, suggesting that HIF1α is involved in early stages of SI [29]. Therefore, HIF1α may play critical roles in early negative regulation of inflammation resolution by promoting neutrophil survival and migration, and delaying the switch of macrophages to anti-inflammatory phenotypes [30]. Further, activation of HIF1α was found to delay inflammation resolution by reducing neutrophil apoptosis and reverse neutrophil migration [31]. The 2 other short-listed genes in *PNR1 Prkch* and *Fntb* have a less defined role in inflammatory responses. We further sought variations in *PNR1* candidate genes, particularly *Hif1α*, that may influence NR in SI impacting the course of resolution.

Potential SNPs that may influence expression and function of Hif1α were analyzed in the WebQTL registry. *Hif1α* contains 197 known SNPs between parents A and B, with one synonymous SNP in exon 6 and the remaining are in introns. Synonymous SNPs also have the ability to affect the final protein product [32]. These seemingly benign differences can alter function or expression of the protein through translational regulation, differential splicing, miRNA binding, mRNA and protein folding, and post translational modification [32]. QTL analysis using the AXB-BXA panel reveals relevant loci based on sequence variation between the two parental strains. Therefore, identified QTLs may point at a gene whose protein sequence remains unchanged regardless of differences in expression levels [33].

QTL mapping is usually made based on as many RI strains as practically possible [13]. This study had 18 available strains including 16 of the AXB-BXA strains and the 2 parental strains, which was sufficient to highlight 3 QTLs linked to NR one of which was significantly linked to the trait under study. This may indicate that the causative gene at this QTL has a strong genetic influence on this trait. This conclusion is supported by the heritability estimate that over half of the variation across the strains could be explained by the effect of genetic factors. It is possible that inclusion of more strains could lead to *PNR2* and *PNR3* becoming significant QTLs and perhaps unraveling additional QTLs. Future studies could be done using other GRPs such as the popular BXD RI murine panel. This group of inbred mice is the product of the cross between C57BL/6J X DBA/2J and consists of ~160 strains [13]. Preliminary experiments would ascertain if the C57BL6/J and DBA2/J parental strains contrast on NR [13].

Gene candidacy was prioritized in part based on the current knowledge of their biological function. Thus, well-studied genes are more likely to be detected in our prioritization algorithm as relevant to our trait. Therefore, some of those genes less well studied may have a functional relevance to NR that is yet to be characterized. For example, RIKEN cDNA 1200003C05 was high on the gene expression list but its function in currently not known. Moreover, we set sifting filters that used literature mechanistic and structural relevance, gene expression levels, and the presence of SNPs differing in the parental strains, whereas a different selection of filters may have prioritized other genes than those we selected [13]. Therefore, further studies are needed to validate the candidacy of the genes we prioritized using molecular and genetic approaches that could include using si-RNA, missense and pharmacological treatments to treat mice of the high NR-responsive AXB-BXA strains, or treatments gaining increased NR function using other treatments in mice of the low-responding strains, or testing gene knock-out or knock-in mice *versus* wild-type mice.

In summary, we were able to identify 3 QTLs (*PNR1-3)* that were significantly or suggestively linked to NR in SI. They appear to act independently of each other to affect variation in the NR levels across this strain panel. Candidate genes prioritized in *PNR1* may affect NR levels by conferring cytoprotection in inflammatory sites and may therefore be involved in various immune cell functions. The strongest candidate is *Hif1α*, which may control early neutrophil infiltration and survival into inflamed tissues therefore mediating initiation of resolution programs. Further, the present study supports the view that certain genes, and environmental factors, control NR to a site of inflammation in a site-specific and irritant-specific manner. These findings may help target unregulated pathways of resolution in inflammatory diseases.

## Supporting Information

S1 Table Genes from *PNR2*. Full list of 130 candidate genes located in the *PNR2* QTL on Chr 12. *PNR2* spans a confidence interval of 5.47 Mb (from 87–101.6 and 102.5–105 Mb).
(PDF)

S2 Table Short-listed candidate genes located in *PNR2*. Nineteen genes prioritized through microarray analyses.
(PDF)

S3 Table Genes from *PNR3*. Full list of 96 candidate genes located in the *PNR3* QTL on Chr 16. PNR3 spans a confidence interval of 18.5 Mb (from 56.5–75 Mb).
(PDF)

S4 Table Short-listed candidate genes located within *PNR3*. Six genes were highlighted through microarray analyses.
(PDF)
