# Supplementary material for: Quantitative Trait Loci and Candidate Genes for Neutrophil Recruitment in Sterile Inflammation Mapped in AXB-BXA Recombinant Inbred Mice
Source: PLoS One. 2015 May 5;10(5):e0124117. doi: 10.1371/journal.pone.0124117 (PMC4420501; doi:10.1371/journal.pone.0124117)
Supplement: S1 Table — Full list of 130 candidate genes located in the PNR2 QTL on Chr 12. PNR2 spans a confidence interval of 5.47 Mb (from 87–101.6 and 102.5–105 Mb). (PDF) [file pone.0124117.s001.pdf]

Table S1. **Genes from *PNR2*.** Full list of 130 candidate genes located in the *PNR2* QTL on Chr 12. *PNR2* spans a confidence interval of 5.47 Mb (from 87-101.6 and 102.5-105 Mb).

Gene Symbols			
Batf	Nrp	Galc	LOC100040305
Flvcr2	1810035L17Rik	Gpr65	Cpsf2
0610007P14Rik	Snw1	Kcnk10	Slc24a4
Ttll5	EG266459	5330409N07Rik	Gm46
Tgfb3	EG271022	Spata7	C030009J22Rik
1700019E19Rik	Oog1	Ptpn21	Rin3
Gm805	LOC100039203	Zc3h14	E030047P09Rik
1700020O03Rik	EG435337	9430031K09Rik	Lgmn
D930046M13Rik	Adck1	D230049E03Rik	A930036A04Rik
Esrrb	3200001D21Rik	Eml5	Golga5
Vash1	4930473H19Rik	A930040O22Rik	Chga
Angel1	1700040E09Rik	Ttc8	Itpk1
Gm263	Nrxn3	4930474N09Rik	Moap1
Thsd3	E530011G23Rik	Foxn3	D230037D09Rik
6430527G18Rik	6430597G12Rik	3300002A11Rik	5730410I19Rik
2310044G17Rik	A930035E12Rik	Ttc7b	Btbd7
Zdhhc22	B230375D24Rik	4930556H04Rik	Cox8c
Tmem63c	LOC100039498	1700064M15Rik	9030205A07Rik
Ngb	6330549H03Rik	2610021K21Rik	Prima1
Pomt2	Dio2	Tdp1	Serpina4-ps1
Gstz1	4930544I03Rik	LOC100040041	Asb2
EG625237	1700105G05Rik	Kcnk13	Otub2
Tmed8	4930534B04Rik	4930477G07Rik	9330161L09Rik
LOC100039191	5430427M07Rik	9530050K03Rik	Ddx24
4933437F05Rik	Tshr	Psmc1	D12Ertd647e
AI413782	Gtf2a1	BC002230	Ifi27
9330175H22Rik	Ston2	LOC320288	1810023F06Rik
6720456H09Rik	EG666955	Calm1	EG238395
Ahsa1	LOC100039631	Catsperb	8430415E04Rik
Sptlc2	Sel1l	Tc2n	Serpina10
EG193330	4930559C10Rik	Fbln5	Serpina6
Alkbh1	Flrt2	Atxn3	Serpina1f
	1700019M22Rik		Serpina1b
