# Supplementary material for: Quantitative Trait Loci and Candidate Genes for Neutrophil Recruitment in Sterile Inflammation Mapped in AXB-BXA Recombinant Inbred Mice
Source: PLoS One. 2015 May 5;10(5):e0124117. doi: 10.1371/journal.pone.0124117 (PMC4420501; doi:10.1371/journal.pone.0124117)
Supplement: S2 Table — Nineteen genes prioritized through microarray analyses. (PDF) [file pone.0124117.s002.pdf]

Table S2. Short-listed candidate genes located in *PNR2*. Nineteen genes prioritized through microarray analyses.

Index	Symbol	Gene	Microarray BM vs. PE FC*	Microarray Blood vs. PE FC*
1	*Kcnk13*	Potassium channel, subfamily K, member 13	7.15	
2	*Asb2*	Ankyrin repeat and SOCS box-containing 2	5.21	
3	*Lgmn*	Legumain	5.00	
4	***Batf***	**Basic leucine zipper transcription factor, ATF-like**	4.84	5.40
5	*2610021K21Rik*	RIKEN cDNA 2610021K21 gene	-3.35	
6	*BC002230*	cDNA sequence BC002230	-2.41	
7	*Otub2*	OTU domain, ubiquitin aldehyde binding 2	-2.31	
8	***Zc3h14***	**Zinc finger CCCH type containing 14**	-2.23	-2.02
9	*Ttc7b*	Tetratricopeptide repeat domain 7B	-2.04	
10	*4930544I03Rik*	RIKEN cDNA 4930544I03 gene	-1.95	
11	*4930534B04Rik*	RIKEN cDNA 4930534B04 gene	-1.95	
12	*Tmed8*	transmembrane emp24 domain containing 8	-1.94	
13	*Ttll5*	tubulin tyrosine ligase-like family, member 5	-1.77	
14	*Pomt2*	protein-O-mannosyltransferase 2	1.74	
15	*Ddx24*	DEAD (Asp-Glu-Ala-Asp) box polypeptide 24	1.72	
16	***6430527G18Rik***	**RIKEN cDNA 6430527G18 gene**	1.66	-3.08
17	*Gpr68*	G protein-coupled receptor 68		-3.21
18	*0610007P14Rik*	RIKEN cDNA 0610007P14 gene		-2.13
19	*Gpr65*	G-protein coupled receptor 65		2.36

BM – Bone Marrow; PE – Peritoneum; FC – Fold Change * FC significant at $p<0.05$.
Bolded genes show significance with both microarray data sets.
