# Supplementary material for: Quantitative Trait Loci and Candidate Genes for Neutrophil Recruitment in Sterile Inflammation Mapped in AXB-BXA Recombinant Inbred Mice
Source: PLoS One. 2015 May 5;10(5):e0124117. doi: 10.1371/journal.pone.0124117 (PMC4420501; doi:10.1371/journal.pone.0124117)
Supplement: S3 Table — Full list of 96 candidate genes located in the PNR3 QTL on Chr 16. PNR3 spans a confidence interval of 18.5 Mb (from 56.5–75 Mb). (PDF) [file pone.0124117.s003.pdf]

Table S3. Genes from *PNR3*. Full list of 96 candidate genes located in the *PNR3* QTL on Chr 16. PNR3 spans a confidence interval of 18.5 Mb (from 56.5-75 Mb).

**Gene Symbols**			
Abi3bp	Olfr176	Olfr206	Pit1-rs1
Tmem45a	Olfr177	Olfr207	Pou1f1
2310005G13Rik	Olfr178	Olfr208	Chmp2b
Tomm70a	4933431I19Rik	Olfr209	4933411O13Rik
Nit2	Olfr180	Gabrr3	2900041H08Rik
Tbc1d23	Olfr181	1700022E09Rik	9330155M09Rik
D030022P07Rik	Olfr183	Mina	4930428D20Rik
Tmem30c	Olfr186	BC043118	Speer2
2610528E23Rik	Olfr187	Arl6	Gbe1
4921517D16Rik	Olfr190	A930013N22Rik	4930423O20Rik
4631422O05Rik	Olfr191	4930547E14Rik	D16Ertd519e
Col8a1	Olfr192	9330168O09Rik	4931420L22Rik
C130020P16Rik	Olfr193	Epha6	4930529F21Rik
4930461C15Rik	Olfr194	EG224276	4930567J20Rik
Dcbld2	Olfr195	Nsun3	3110049I03Rik
St3gal6	Olfr196	Arl13b	8030451O07Rik
Gm813	Olfr197	Stx19	Robo1
E330017A01Rik	Olfr198	Pros1	D230034L24Rik
Cpox	Olfr199	Epha3	C030046M01Rik
Gpr15	Olfr201	4930453N24Rik	4930500H12Rik
Cldnd1	Olfr202	Zfp654	2310066N15Rik
Olfr172	Olfr203	2810421E14Rik	Robo2
Olfr173	Olfr204	Cggbp1	9330209N08Rik
Olfr174	Olfr205	Htr1f	2510042H03Rik
