# Supplementary material for: Quantitative Trait Loci and Candidate Genes for Neutrophil Recruitment in Sterile Inflammation Mapped in AXB-BXA Recombinant Inbred Mice
Source: PLoS One. 2015 May 5;10(5):e0124117. doi: 10.1371/journal.pone.0124117 (PMC4420501; doi:10.1371/journal.pone.0124117)
Supplement: S4 Table — Six genes were highlighted through microarray analyses. (PDF) [file pone.0124117.s004.pdf]

Table S4. Short-listed candidate genes located within *PNR3*. Six genes were highlighted through microarray analyses.

Index	Symbol	Gene	Microarray BM vs. PE FC*	Microarray Blood vs. PE FC*
1	*Pros1*	Protein S (alpha)	7.44	
2	***St3gal6***	**ST3 beta-galactoside alpha-2,3-sialyltransferase 6**	4.41	4.78
3	*Arl13b*	ADP-ribosylation factor-like 13B	2.98	
4	***2610528E23Rik***	**RIKEN cDNA 2610528E23 gene**	-2.29	-1.83
5	*Gbe1*	Glucan (1,4-alpha-), branching enzyme 1	-1.89	
6	*Tbc1d23*	TBC1 domain family, member 23		2.43

BM – Bone Marrow; PE – Peritoneum; FC – Fold Change * FC significant at $p<0.05$. Bolded genes show significance with both microarray data sets.
